# Editorial to the Special Issue “Molecular and Cellular Mechanisms of CVD: Focus on Atherosclerosis”

**DOI:** 10.3390/biomedicines12092148

**Published:** 2024-09-23

**Authors:** Nikita G. Nikiforov

**Affiliations:** 1 Laboratory of Angiopathology, The Institute of General Pathology and Pathophysiology, 125315 Moscow, Russia; nikiforov.mipt@googlemail.com; 2 Center for Precision Genome Editing and Genetic Technologies for Biomedicine, Institute of Gene Biology, Russian Academy of Sciences, 119334 Moscow, Russia; 3 Engelhardt Institute of Molecular Biology, Russian Academy of Sciences, 119991 Moscow, Russia

The current Special Issue, “Molecular and Cellular Mechanisms of CVD: Focus on Atherosclerosis”, is dedicated to exploring the various mechanisms involved in atherogenesis. Modern theories of atherosclerosis offer comprehensive explanations for many of its stages, and rather than conflicting, these theories complement one another [1,2,3]. In this editorial, I aim to describe the main results of the articles in this Special Issue and discuss some of the mechanisms of atherogenesis.

The lipid theory posits that atherosclerosis results from lipid accumulation in the vascular wall, a process triggered by the chemical modification of circulating low-density lipoproteins (LDL). However, oxidation alone is not sufficient to cause the accumulation of intracellular cholesterol; the levels of oxidized LDL in the blood of atherosclerotic patients have not been found to be significantly elevated [4,5]. Subsequent research revealed that LDL from the blood of atherosclerotic patients exhibited reduced levels of sialic acid [6]. Furthermore, in vitro desialylated LDL showed increased density, a greater negative charge, decreased size, and a propensity for spontaneous self-association [7]. This multiply modified LDL induced the formation of lipid-laden foam cells in vitro. The exact agents responsible for LDL desialylation and their role in atherosclerosis remain unclear. It is possible that neuraminidases, which possess trans-sialidase activity and can desialylate LDL particles, may be involved in this process [8,9,10].

Publications proposing new theories on atherogenesis are extremely encouraging. Sesorova et al. investigated a novel mechanism of LDL desialylation and presented an original hypothesis [11]. Their research showed that administering large amounts of plant lipids to the stomachs of rats led to the formation of enlarged chylomicrons within the endolysosomes of enterocytes in their small intestines. These altered chylomicrons were retained in the endolysosomes and came into contact with neuraminidase type I, which is capable of cleaving sialic acid. Notably, lipid overload was associated with increased binding of circulating LDL to the basement membrane of endothelial cells. This suggests that high fat intake might lead to the desialylation of chylomicrons, which are then converted into desialylated LDL. Combined with increased endothelial permeability in areas of arterial bifurcation prone to atherosclerosis, the accumulation of lipids influenced by desialylated LDL could potentially act as a trigger for atherosclerosis.

Thus, one potential cause of atherosclerosis may be related to the peculiarities of lipid metabolism in individuals with a high-fat diet. Poledne et al. evaluated the role of diet in the development of atherosclerosis [12]. They presented epidemiological data showing a significant decline in mortality among men in the Czech Republic between 1988 and 1992. This period was unique due to a notable change in food prices in early 1991, which halved butter consumption and increased the consumption of vegetable oils. This dietary shift was associated with a decrease in non-high-density lipoprotein cholesterol levels among the population. Histological examinations also revealed a reduction in the number of proinflammatory macrophages in adipose tissue during this time. Interestingly, the proinflammatory phenotype of macrophages was directly related to the levels of palmitate and palmitoleate in cell membranes and inversely correlated with the levels of n-3 fatty acids. These findings suggest that palmitate has pro-inflammatory properties and can induce an inflammatory response in its free form [13]. Unfortunately, palmitate is still widely used in the food industry for thermal stabilization of products. Moreover, dietary patterns were found to influence not only blood lipid composition but also immune cell activity. This aligns with observations that lipid accumulation in macrophages, influenced by modified LDL, is accompanied by significant changes in the expression of inflammatory genes [14].

Atherosclerosis is known to be accompanied by chronic local inflammation [15]. Certain inflammatory molecules may serve as reliable markers for the early stages of atherosclerosis. For example, Loch et al. demonstrated the prognostic value of leucine-rich α2-glycoprotein in serum for detecting early diastolic dysfunction [16]. However, it remains unclear why the inflammatory response in the vascular wall fails to resolve and becomes chronic. Previous research has shown that proinflammatory macrophages associated with unstable lesions are of hematogenous origin [17].

On one hand, the infiltration of immune cells and lipids from the bloodstream into the vascular wall may result from endothelial dysfunction [18]. Endothelial dysfunction is characterized by increased permeability, reduced proliferative potential, and heightened secretion of cytokines and chemokines. Ponasenko et al. identified polymorphisms in genes related to vitamin D metabolism and endothelial homeostasis in patients with coronary artery disease [19]. Additionally, Poston et al. observed increased levels of malondialdehyde (MDA) adducts and heat shock protein 60 (HSP60) on the endothelium of atherosclerotic plaques, which may promote monocyte adhesion [20]. Endothelial cell injury and/or dysfunction appears to be one of the initial events in atherogenesis.

On the other hand, increased immune cell infiltration into the vascular wall may result not only from endothelial dysfunction but also from changes within the immune cells themselves. Notably, proinflammatory alterations in blood monocytes associated with atherosclerosis can occur while the cells are still in circulation [21]. We have previously shown that circulating monocytes from patients with preclinical atherosclerosis exhibit impaired tolerance to lipopolysaccharide (LPS), evidenced by increased secretion of monocyte chemoattractant protein 1 (MCP-1) [22]. Monocyte tolerance to LPS is a normal defense mechanism against hyperinflammation, and its impairment may contribute to the chronicity of inflammation. Continuous elevated secretion of MCP-1, a chemoattractant for monocytes, may further exacerbate inflammation by attracting additional immune cells [23].

Moreover, atherosclerosis is associated with changes not only in monocyte function but also in the composition of their subpopulations. Kologrivova et al. demonstrated an increase in the intermediate monocyte subpopulation (CD14^++^CD16^+^) in patients with coronary artery disease and type 2 diabetes mellitus [24,25]. These findings suggest that alterations in monocyte subpopulation composition may contribute to the observed proinflammatory state of these cells. Earlier research by Ong et al. identified signs of cellular senescence in CD16^+^ monocytes [26]. Conversely, CD16^+^ monocytes have also been shown to roll along the endothelium, possibly participating in endothelial repair [27]. These observations may not be contradictory, suggesting that CD16^+^ monocytes (whether non-classical or intermediate) play a significant role in the chronicity of inflammation and exhibit increased adhesion to the endothelium. However, the underlying mechanisms responsible for the shift in monocyte subpopulation ratios in the blood remain unclear. It is known that increased proinflammatory activity of monocytes in atherosclerosis can originate at the level of progenitor cells in the bone marrow [28]. It is likely that these changes also influence the differentiation of monocytes into various subpopulations.

This Special Issue features two reviews. It is well established that cardiovascular complications arising from the development of atherosclerosis are the leading cause of death in individuals with type 2 diabetes mellitus. In their review, Nedosugova et al. explore the critical roles of oxidative stress and chronic inflammation in the progression of atherosclerosis associated with type 2 diabetes mellitus [29]. Meanwhile, Ng et al. discuss emerging biomarkers of oxidative stress and inflammation in their new review [30].

Despite significant advances in extending life expectancy and improving quality of life in old age, effective methods for inducing regression of atherosclerosis remain elusive. The publications in this Special Issue address various facets of atherogenesis, including lipid metabolism disorders, LDL modifications, endothelial dysfunction, and changes in immune cell function. It is now evident that these aspects are interconnected in complex ways. Endothelial dysfunction not only increases permeability but also enhances proinflammatory signaling and monocyte adhesion. Unbalanced nutrition can lead to desialylation and subsequent modifications of LDL particles, such as increased density, decreased size, increased negative charge, and self-association. Monocytes and macrophages attracted to the inflammation site engage in the uptake of LDL particles infiltrated into the vascular wall, transforming into foam cells. Notably, monocytes in circulation already exhibit a pro-inflammatory status and tend to secrete excess MCP-1, attracting additional monocytes to the inflammation site. Furthermore, changes in the expression of inflammatory genes can be observed at the precursor level in the bone marrow. The reasons and mechanisms behind pro-inflammatory changes in circulating monocytes, LDL particle modifications, and endothelial dysfunction remain unclear. Continued research is essential to develop a unified theory of atherosclerosis that accounts for its multifactorial nature.

I am pleased that this Special Issue features such remarkable research. I would like to thank the authors for their invaluable contributions, which bring us closer to overcoming atherosclerosis. Many thanks also to the reviewers and the *Biomedicines* team.
